# Supplementary figures and images for: Potyvirus HcPro Suppressor of RNA Silencing Induces PVY Superinfection Exclusion in a Strain-Specific Manner
Source: Int J Mol Sci. 2025 Dec 1;26(23):11644. doi: 10.3390/ijms262311644 (PMC12692455; doi:10.3390/ijms262311644)

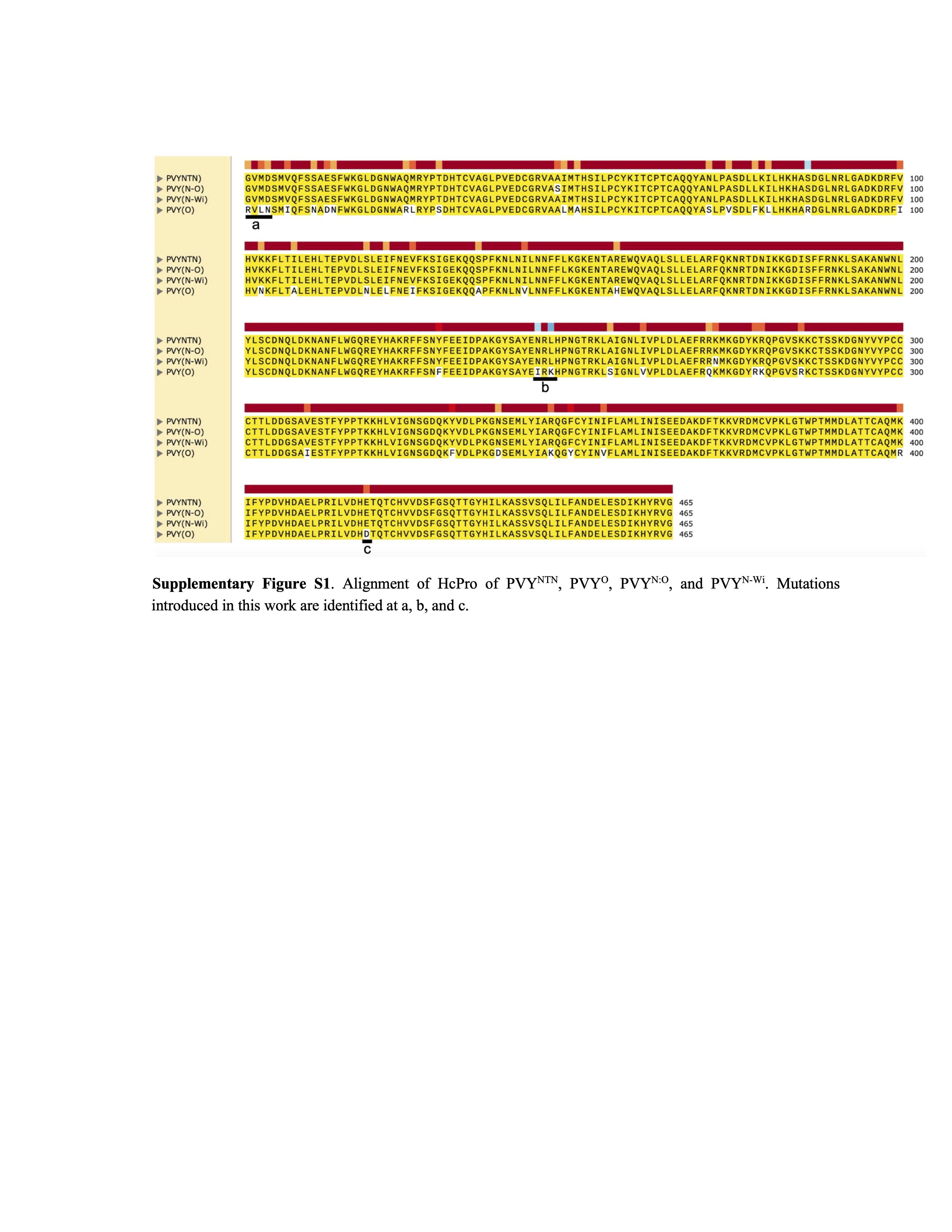

Supplement: Supplementary file 1 [file ijms-26-11644-s001.zip › Figure S1.tiff]
